# Neutrophil Heterogeneity: Molecules to Cellular Behavior

**DOI:** 10.3390/life16040696

**Published:** 2026-04-21

**Authors:** Jonghee Lee, Jingu Lee

**Affiliations:** 1 Department of Physiology, School of Medicine, Pusan National University, Yangsan 50612, Republic of Korea; 202687126@pusan.ac.kr; 2 Research Institute for Convergence of Biomedical Science and Technology, Pusan National University Yangsan Hospital, Yangsan 50612, Republic of Korea

**Keywords:** neutrophils, heterogeneity, single cell analysis, intravital imaging

## Abstract

Neutrophils constitute the largest fraction of total circulating leukocytes in humans and mediate early innate immune responses. Although they are often considered a uniform population of short-lived immune cells, emerging evidence from single-cell RNA sequencing and high-dimensional flow cytometry has revealed that neutrophils are functionally and phenotypically heterogeneous in both healthy and pathological conditions. However, a critical gap is how molecularly defined neutrophil states translate into distinct spatiotemporal behaviors in vivo. This review summarizes our current understanding of the molecular signatures underlying neutrophil heterogeneity and explores the functional in vivo behaviors in various diseases, including cancer, sepsis, and ischemic stroke. We also discuss the potential of intravital imaging to bridge the gap between static molecular profiling and dynamic cellular behavior, offering a comprehensive view of the functional heterogeneity of neutrophils.

## 1. Introduction

Neutrophils play a central role in the immediate innate immune response and rapidly migrate to sites of infection or tissue injury to perform various functions, including degranulation (granule secretion), phagocytosis, reactive oxygen species (ROS) generation and neutrophil extracellular trap (NET) formation [1,2,3]. These responses are important for host defense; however, when overactive or dysregulated, neutrophils can exacerbate inflammation and contribute to tissue damage, thereby contributing to a wide range of pathological conditions, including cancer [4,5], sepsis [6,7] and ischemic stroke [8,9,10]. Previously, neutrophils were considered to be short-lived, terminally differentiated immune cells with relatively limited phenotypic variation [11]. However, a growing body of evidence supports heterogeneity and plasticity across developmental stages, activation states, and tissue contexts. Single-cell RNA sequencing has identified transcriptionally distinct neutrophil states and maturation trajectories in both healthy and pathological conditions [12,13,14], whereas high-dimensional flow cytometry has identified disease-associated neutrophil states [15,16]. Despite these molecular insights, a critical limitation remains: the disconnection between static molecular profiles and in vivo cellular behaviors. Under inflammatory conditions, neutrophil recruitment to inflamed tissues is a tightly orchestrated multi-step process involving rolling, arrest, crawling and transendothelial migration (TEM), which are regulated by interactions with endothelial adhesion molecules [17]. Although omics approaches provide detailed snapshots of cellular states, they often fail to capture the real-time spatiotemporal dynamics in vivo.

**Figure 1:**
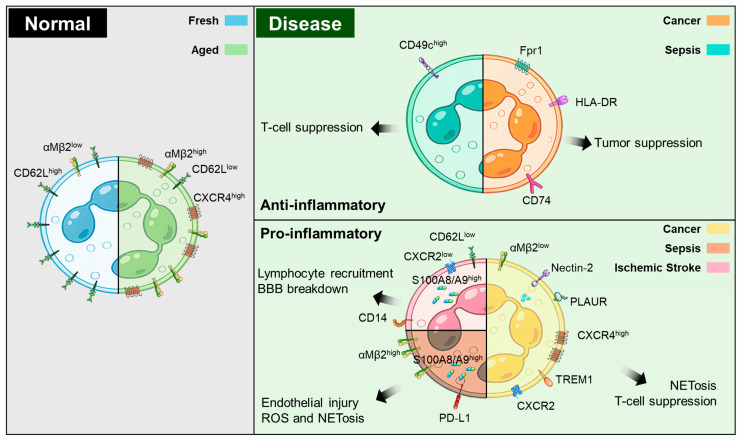
Neutrophil heterogeneity under physiological and pathological conditions. Under normal conditions, circulating neutrophils transition from ‘fresh’ (CD62L^high^αMβ2^low^CXCR4^low^) to ‘aged’ (CD62L^low^αMβ2^high^CXCR4^high^) populations. Under pathological conditions such as cancer, sepsis, and ischemic stroke, neutrophils further diversify into disease-associated states defined by distinct transcriptional and surface-marker signatures. Anti-inflammatory or protective neutrophils in cancer and sepsis are characterized by representative markers linked to host-protective or anti-tumor functions. In contrast, pro-inflammatory neutrophils in cancer, sepsis, and ischemic stroke are associated with representative markers linked to immunosuppression, endothelial injury, BBB breakdown, ROS production, and NETosis. Overall, this schematic highlights how neutrophil heterogeneity can be linked to either protective or pathogenic functions across physiological and pathological conditions.

Intravital microscopy (IVM) has emerged as a critical tool for bridging this gap. Unlike static analyses from omics, IVM allows for in vivo real-time visualization and quantification of immune cell behavior under pathological conditions [18,19]. Using reporter mice or in vivo fluorescent labeling strategies, IVM enables real-time monitoring of neutrophils within living tissues at a single-cell resolution. This approach allows the quantitative analysis of spatiotemporal behaviors such as adhesion, crawling, TEM, and interactions with resident cells in the local microenvironment [20]. Recent advances in imaging analysis have enabled the linking of in vivo behavioral data to distinct cell populations, providing behavioral phenotypes at the single-cell level [21,22]. Thus, integrating single-cell molecular analysis with intravital imaging offers a robust platform for comprehensively understanding neutrophil heterogeneity in vivo (Figure 1). This review discusses the potential applications of IVM to elucidate the link between molecular heterogeneity and cellular behavior, thereby offering an integrated perspective on neutrophil function from in vitro profiling to in vivo dynamics.

## 2. Molecular Signatures to Cellular Behaviors Under Healthy Conditions

Granulocyte colony-stimulating factor (G-CSF) promotes bone marrow granulopoiesis, thereby facilitating the egress of mature neutrophils into peripheral blood [23,24]. The CXCL12-CXCR4 pathway functions as a major retention signal that maintains mature neutrophils within the bone marrow, whereas CXCL1 and CXCL2, acting through CXCR2, promote neutrophil egress into the blood [25,26]. G-CSF promotes neutrophil mobilization by reducing CXCR4 expression in the marrow [27]. A recent study reported that under homeostatic circadian conditions in mice, fresh neutrophils are released from zeitgeber time (ZT)17 to ZT5, and aged neutrophils are cleared from ZT5 to ZT13 [28]. Here, the ZT is defined relative to the light–dark cycle, with ZT0 indicating lights on and ZT12 indicating lights off. Because mice are nocturnal, whereas humans are diurnal, the physiological meaning of a given ZT value differs between the two species; under conventional light-dark conditions, ZT0-12 broadly corresponds to the active phase in humans but to the inactive phase in mice [29]. Phenotypically, circulating neutrophils can be defined by CD62L and CXCR4 expression: CD62L^high^CXCR4^low^ cells represent a “fresh” population released from the bone marrow, whereas CD62L^low^CXCR4^high^ cells are defined as “aged” blood neutrophils, reaching their highest proportion at ZT5 [28]. Mechanistically, circadian regulation of neutrophils depends on both external and internal cues. Freshly circulating neutrophils undergo a Bmal1-dependent intrinsic aging program in which autocrine CXCL2-CXCR2 signaling promotes the transition from fresh to aged states [29]. Notably, aged neutrophils exhibit increased surface levels of CD11b and CD49d, suggesting adhesive/trafficking features [30]. Additionally, aged neutrophils migrate back to the bone marrow via CXCL12-CXCR4 signaling, where they are subsequently engulfed and cleared by resident CD169^+^ macrophages, this clearance supporting circadian oscillations in neutrophil homeostasis [28].

To investigate neutrophil aging in vivo, IVM of inflamed microvessels identified a CD62L^low^ neutrophil state with enhanced Mac-1 activation, consistent with microbiome-driven TLR-MyD88 signals that promote the emergence and activation of aged neutrophils [31]. Recent intravital imaging in a murine stroke model showed that at ZT5 (the inactive phase), aged neutrophils were associated with increased microvascular stalling and reduced collateral perfusion, suggesting that time-of-day-dependent neutrophil-microvessel interactions impair flow and thereby worsen stroke injury [32]. Additionally, emerging evidence suggests that circadian dysregulation affects tumor-associated neutrophil populations. In experimental metastatic cancer models, CXCR4^high^CD62L^low^ aged neutrophils were enriched in association with disrupted neutrophil circadian homeostasis, indicating that tumors can perturb physiological neutrophil aging and selectively expand the metastasis-associated state [33]. In colorectal cancer, the disruption of the epithelial clock is associated with increased neutrophil recruitment and myeloid-derived suppressor cell (MDSC) accumulation. Notably, the associated neutrophilic state was characterized by the expression of S100a8, S100a9, and CXCR2, and the enrichment of MDSC-related markers, such as Ifitm1, Wfdc17, Irg1, and Arg2, together with increased ROS and PD-L1, consistent with an immunosuppressive phenotype [34].

## 3. Molecular Signatures to Cellular Behaviors Under Pathological Conditions

Under pathological conditions, such as cancer, sepsis, and ischemic stroke, neutrophils respond and migrate to inflamed sites, exhibiting heterogeneous phenotypes. Here, we discuss the current understanding of molecular signatures across these diseases and how functional in vivo behaviors can be investigated using intravital imaging. A deeper understanding of the relationship between the phenotypic states and in vivo behavioral characteristics of neutrophils provides a comprehensive view of neutrophil functional heterogeneity and contributes to the identification of novel therapeutic targets.

### 3.1. Cancer

Tumor-associated neutrophils (TANs) are broadly described in anti-tumorigenic N1 and pro-tumorigenic N2 states. TGF-β signaling in the tumor microenvironment promotes polarization toward a tumor-supportive N2 state, whereas blockade of TGF-β shifts TANs toward an N1 state. Functionally, N1 TANs exhibit enhanced cytotoxic activity against tumor cells, exerting an anti-tumorigenic function, whereas N2 TANs are immunosuppressive, thereby promoting tumor progression. In addition, N1-polarized TANs enhance CD8^+^ T cell activation, whereas N2-polarized TANs suppress anti-tumor T cell responses [35,36]. In parallel, pseudo-time and trajectory analyses further suggested that TGF-β/SMAD3 signaling maintains TANs in an N2-like state, whereas Smad3 deficiency or pharmacologic inhibition shifts TANs toward an N1-like state, with increased neutrophil infiltration and tumor regression [37]. Consistent with this, in lung adenocarcinoma, a miR-941/FOXN4/TGF-β feedback loop has been proposed to promote N2-like TAN polarization and tumor progression, linking TAN-associated miRNA activity to FOXN4 suppression and enhanced TGF-β signaling in tumor cells [38]. In cancer, neutrophils appear to undergo stage-dependent reprogramming, transitioning from early polymorphonuclear myeloid-derived suppressor cells (PMN-MDSC)-like neutrophils, characterized by enhanced migratory capacity and metabolic activation, to later PMN-MDSCs/activated PMN-MDSCs with enhanced immunosuppressive activity [39,40]. Moreover, lineage-defining transcription factors, such as SOX2 and NKX2-1 can shape the tumor immune microenvironment in non-small cell lung cancer (NSCLC) by regulating TAN recruitment. In this context, TANs exhibit N2-like features, including SiglecF expression and increased ROS-related signatures, and neutrophil depletion reduces squamous tumor burden, suggesting that TANs contribute to squamous tumor progression [41]. Interestingly, the modulation of neutrophil states in cancer may be a potential complementary strategy for strengthening anti-cancer immunotherapy. For example, trained innate immunity can reprogram granulopoiesis, thereby shifting neutrophils toward an anti-tumor phenotype. In particular, β-glucan preconditioning induces transcriptional and epigenetic remodeling at the level of granulocyte-monocyte progenitors, generating neutrophils with enhanced ROS-dependent tumor-killing activity that suppresses tumor growth [42]. In resistant head and neck squamous cell carcinoma (HNSCC), Sox9^+^ tumor cells were enriched and promoted the apoptosis of Fpr1^+^ neutrophils. This loss of Fpr1^+^ neutrophils was associated with reduced infiltration and cytotoxic activity of CD8^+^ and γδ T cells, ultimately contributing to resistance to combined anti-LAG-3 and anti-PD-1 immunotherapy [43].

In addition to the conventional N1/N2 TAN concept, recent single-cell transcriptomic and proteomic studies have revealed the cancer-specific neutrophil states. Single-cell transcriptomic profiling of human and mouse lung cancers revealed that TANs represent a heterogeneous population, including canonical neutrophil-like states, tumor-enriched pro-tumor states, and distinct type I interferon-responsive neutrophil states, supporting the state-based classification of TANs [44]. Integrated single-cell analysis across 17 cancer types identified 10 distinct neutrophil states [45]. Among these, anti-tumor or protective states include HLA-DR^+^CD74^+^ neutrophil states, which exhibit an MHC class II/antigen-presenting signature and are associated with better clinical outcomes. In gastric cancer, single-cell transcriptomic analysis has identified CD44^−^CXCR2^−^ tumor-specific neutrophils (tsNeus) as a distinct neutrophil state, including a CD54^+^ tsNeu state with anti-tumor activity linked to reduced tumor progression and improved survival [46]. Additionally, neutrophil-induced pyroptosis involving ELANE^+^ neutrophils, NETs, and CASP1-associated pyroptotic signaling is associated with increased CD8^+^ T cell activation [47].

In contrast, immunosuppressive or tumor-promoting states include Nectin2-expressing TAN states associated with CD8^+^ T-cell dysfunction/exhaustion and cell–cell adhesion, suggesting a role for this neutrophil state in an immunosuppressive tumor microenvironment [48], a CXCR4^high^ neutrophil state in hepatoblastoma characterized by ferroptotic signatures together with T-cell suppressive features and chemotactic behavior [49], a PLAUR^+^ neutrophil state in hepatocellular carcinoma linked to reduced CD8^+^ T-cell infiltration and macrophage-driven immunosuppression, and migration [50]. Tumor-enriched CD83^+^ TANs characterized by the upregulation of CXCR4 and downregulation of CXCR2/SELL, consistent with a senescent-like phenotype and immunosuppressive signatures, and associated with poor prognosis across various cancer types [51]. Similarly, in patients with NSCLC, circulating neutrophil-platelet aggregates (NPAs) have been identified in peripheral blood, and a platelet gene-high neutrophil cluster is associated with degranulation, chemotaxis, and TEM, indicating an activated and tumor-promoting circulating neutrophil state [52]. Importantly, the NPA-associated neutrophil signature was correlated with poor prognosis across multiple solid tumor types, supporting its potential as a prognostic biomarker.

Pro-inflammatory states include an immature CD10^−^ neutrophil (CD66b^high^CD11b^low^) state enriched in the low-density neutrophil fraction in patients with glioblastoma (GBM) [53]. Single-cell proteomic analysis demonstrated that GBM TANs comprise multiple functional states, including ARG1-linked immunosuppressive/angiogenic signatures and degranulation/NETosis-like states. Additional NET-associated states include tumor-enriched PPIF^+^ neutrophil states with a high NET-related score, consistent with NETosis, and a potential link to colorectal cancer progression [54], and CD10^+^TREM1^+^CXCR2^+^ neutrophil states in multiple myeloma that exhibit T-cell suppressive activity and NETosis, and are associated with poor outcomes as well as increased T-cell exhaustion-related signatures [55].

Metabolic states represented by dcTRAIL-R1^+^ terminal T3 states in pancreatic ductal adenocarcinoma are characterized by inflammation-, hypoxia-, and glycolysis-associated gene signatures [56]. Functionally, T3 neutrophils exhibit pro-angiogenic activity that supports tumor growth. Single-cell transcriptomic analysis identified seven neutrophil states, among which OLR1^+^ and MIF^+^ neutrophils were classified as tumor-enriched TAN states [57]. Notably, the MIF^+^ TAN state was associated with hypoxia- and glycolysis-related signatures and was linked to poor clinical outcomes. During cervical cancer progression, single-cell transcriptomic analysis revealed five distinct neutrophil states (N0–N4), among which the N4 state is related to hypoxia, WNT, and inflammatory signaling, contributing to tumor growth [58]. Collectively, these studies indicate that neutrophil states in cancer are linked to distinct functional outcomes and behavioral features, including anti-tumor activity, immunosuppression, chemotaxis, TEM, NETosis, and pro-angiogenic metabolic adaptation (Table 1).

IVM has been used to observe neutrophil dynamics in tumor models in vivo. In a cutaneous melanoma model, repetitive UVB irradiation induces skin inflammation and increases neutrophil infiltration, promoting melanoma angiotropism and enhancing metastatic dissemination [59]. In an HNSCC-like murine tumor model, intravital two-photon microscopy revealed that intratumoral TANs remained within the tumor core for longer periods, whereas peritumoral TANs exhibited increased motility and migration [60]. In a transplanted murine head and neck cancer model, two-photon microscopy visualized in vivo interactions between neutrophils and T cells in tumor-draining lymph nodes (TDLNs) [61]. IFNAR1 deficiency enhanced neutrophil accumulation in the TDLN but resulted in impaired neutrophil-T cell interactions and increased tumor progression. In the subcutaneous MC38-cEGFR tumor model, intravital imaging revealed that FcαRI-TNF increased tumor-infiltrating neutrophils [62]. Overall, these IVM studies demonstrate that intravital imaging can be used to monitor neutrophil infiltration/accumulation, spatial retention versus migratory behavior within the tumor microenvironment, and neutrophil-T cell interactions, thereby highlighting in vivo behavioral features that may relate to the molecularly defined neutrophil states described above. However, the mechanism by which each transcriptionally defined neutrophil state corresponds to distinct patterns of tumor infiltration, migration, and immune cell interactions in vivo remains unclear. Further integration of intravital imaging with transcriptomic profiling is important to define how specific neutrophil states contribute to tumor progression and functions within the tumor microenvironment.

### 3.2. Sepsis

Sepsis is a leading cause of global mortality and is associated with organ dysfunction [63]. Septic conditions induce dynamic host inflammatory responses, typically defined as acute hyperinflammation and subsequent persistent immunosuppression, which may contribute to mortality through impaired clearance of secondary infections [64,65]. In this context, neutrophils in sepsis are increased due to delayed apoptosis and impaired clearance, as well as the release of immature cells from the bone marrow [66,67]; however, their functional capacity is markedly impaired, including impaired bacterial clearance, attenuated ROS generation, and defective recruitment to sites of infection [68]. Moreover, depending on the stage of sepsis, neutrophils exhibit either pro-inflammatory functions [66] or immunosuppressive phenotypes characterized by IL-10 production and T-cell suppressive activity [69,70]. These functional distinctions may provide a useful framework for understanding molecularly defined neutrophil states in sepsis.

Recent single-cell analyses have suggested that the molecularly defined neutrophil states in sepsis can be broadly organized into anti-inflammatory/protective, pro-inflammatory/NET-associated, and immunosuppressive states. An anti-inflammatory or protective state is represented by a C1q-upregulating CD49c^high^ neutrophil state associated with improved survival [71]. In contrast, pro-inflammatory states include an olfactomedin-4 (OLFM4)-positive neutrophil state [72], and flow cytometric analyses in adult patients with sepsis also identified circulating OLFM4^+^ neutrophils, which were associated with 60-day mortality [73]. These OLFM4-positive states are associated with poor clinical outcomes; however, their specific in vivo functions remain unclear. An animal study using a cecal ligation and puncture (CLP)-induced sepsis model revealed a pro-inflammatory Ly6G^+^ CD11b^high^ low-density neutrophil state, which displayed enhanced production of pro-inflammatory mediators and increased NET formation, thereby contributing to inflammatory tissue injury [74]. Additionally, S100A8/A9^high^ neutrophils promote endothelial mitochondrial dysfunction and PANoptosis [75], linking this state to endothelial injury and microvascular damage. An immunosuppressive state was defined as IL1R2-expressing neutrophils representing an immature and immunosuppressive state linked to poor clinical outcomes [76,77]. Furthermore, PD-L1-expressing neutrophils in sepsis have been reported to contribute to immunosuppression by inhibiting T-cell responses, and are linked to poor clinical prognosis [78]. Integrative analyses in human and murine models further delineated these PD-L1^+^ cells as an immature, transcriptionally distinct state that suppresses T-cell activation and can additionally promote endothelial injury and pyroptosis [15,79,80,81,82], consistent with the molecular state of both immunosuppressive and endothelial-injurious functions. Although these studies defined molecularly distinct neutrophil states in sepsis (Table 2), linking these states to their corresponding in vivo spatiotemporal behaviors requires approaches such as intravital microscopy.

Recently, IVM has been widely used to visualize neutrophil responses within inflamed lung tissues in vivo. Several IVM studies using lipopolysaccharides (LPS)-induced acute lung injury or sepsis models have demonstrated pulmonary endothelial barrier disruption accompanied by functional microvascular dead space [83], accumulation of capillary-entrapped Mac-1-expressing neutrophils associated with impaired microcirculation [19], and increased neutrophil infiltration with NET formation [84,85]. Collectively, these IVM studies highlighted in vivo pathophysiological features of septic neutrophil responses, including endothelial barrier disruption, capillary entrapment with impaired microcirculation, neutrophil infiltration, and NET formation, and conceptually related these phenomena to the molecularly defined neutrophil states described above, particularly the pro-inflammatory/NET-associated and endothelial-injurious states. However, the direct link between these molecular states and distinct in vivo behaviors during sepsis remains unresolved. Further integration of IVM with transcriptomic profiling is important to determine how specific neutrophil states contribute to septic tissue injury in vivo.

### 3.3. Ischemic Stroke

A rapid surge in circulating neutrophils occurs within hours of ischemic stroke onset, and histological evaluations in human patients consistently demonstrate a peak in neutrophil accumulation around days 1–2 post-stroke [86,87]. Clinically, early neutrophilia and an increased neutrophil-to-lymphocyte ratio have been linked to greater stroke severity/infarct volume and poorer outcomes, including higher mortality [88,89]. Previous animal studies have identified pro-inflammatory N1 neutrophils in brain damage and anti-inflammatory N2 neutrophils in an ischemic stroke model for the resolution of inflammation and tissue recovery [90,91,92,93]. In addition, neutrophils release NETs that promote blood–brain barrier (BBB) disruption and thrombosis, thereby contributing to ischemic brain injury [94]. Patients with acute ischemic stroke presenting within 6 h of onset exhibit hyperactivated circulating neutrophils, defined by downregulated L-selectin and upregulated αMβ2 integrin expression, which is linked to stroke severity [95].

Recent studies have suggested that neutrophils exhibit distinct pro-inflammatory and tissue-damaging states under the inflammatory conditions of ischemic stroke. Transcriptomic analyses of purified neutrophils from 38 patients with ischemic stroke showed temporal transcriptional alterations, peaking at 24–48 h post-onset (TP2), including leukocyte extravasation, EIF2 signaling, and TP2-specific IL-1/4/7/8 signaling [96]. In addition, single-cell transcriptomic analysis of aged mice on day 3 post-tMCAO revealed three neutrophil states, among which Neut1 showed upregulated mitochondrial and inflammatory gene programs (including mt-Nd1-4, Il1b, Aif1, and Ly86), consistent with a highly activated neutrophil state infiltrating the ischemic brain and likely contributing to inflammatory tissue injury [97]. Gullotta et al. showed that, in an aged mouse stroke model, CD62L^low^ atypical neutrophils accumulated and were collectively enriched in oxidative stress, phagocytosis, and procoagulant features [98]. Consistently, in elderly patients with stroke, a CD62L^low^ neutrophil state is linked to worse reperfusion and poor functional outcomes. CyTOF and scRNA-seq profiling of aged mice subjected to tMCAO showed that circulating neutrophils shifted toward a late maturation/aging phenotype with decreased CD62L. A brain-enriched neutrophil state (BrNeu2), characterized by CXCR2^low^CD62L^low^ showed senescence, degranulation, and adhesion features linked to increased BBB permeability [99], indicating a recruitment-associated and BBB-disruptive neutrophil state. Moreover, trajectory analysis integrating six blood neutrophil states and six brain granulocyte states from tMCAO-challenged mice indicated that brain granulocytes (Gran2 and Gran3) were recruited from late-stage mature blood neutrophils (Neu3) rather than differentiating locally within the brain [100]. Another study in a mouse model of ischemic stroke also identified a distinct S100A8/A9^high^ neutrophil state, associated with BBB disruption and increased lymphocyte recruitment [101], supporting its role in amplifying vascular inflammation. Interestingly, Vázquez-Reyes et al. reported that neutrophils in the inactive phase (Zeitgeber time 5; ZT5) of a murine stroke model, including pro-inflammatory/degranulation-associated BNc1 and ISG/NETosis-prone BNc4 states, were associated with larger infarcts [32]. In patients, infarct outcomes also showed diurnal variations that correlated with oscillations in NET-related neutrophils, indicating that time-of-day-dependent neutrophils/NETs contribute to stroke outcomes. Furthermore, integrated single-cell transcriptomic analysis identified a CD14-expressing neutrophil state, and flow cytometry confirmed that these cells accounted for approximately 80% of brain neutrophils 24 h after stroke, which is consistent with an activated/mobilized mature neutrophil feature [102]. Collectively, recent single-cell transcriptomic studies have identified distinct neutrophil states characterized by CD62L, S100A8/A9, CXCR2, and CD14 in rodent ischemic stroke models and patients with stroke (Table 3). Although these states share pro-inflammatory and tissue-damaging features, they differ in their functions, including oxidative activity, BBB-disruptive adhesion, leukocyte-recruiting inflammatory signaling, and NET formation. However, the mechanism by which these molecularly defined states translate into distinct spatiotemporal behaviors in the ischemic brain remains unknown. To further investigate the in vivo spatiotemporal behavior of neutrophils, advanced approaches such as intravital imaging could be combined with transcriptomic analyses.

Although single-cell analyses provide static snapshots of neutrophils under inflammatory conditions, IVM is a powerful technique for visualizing neutrophils in vivo. Recent studies using two-photon intravital microscopy revealed increased neutrophil infiltration and reverse TEM (rTEM) in myeloid-specific LysM-GFP mice during LPS-induced neuroinflammation [103], neutrophil-mediated dynamic capillary stalls leading to microvascular flow impairment in LysM-GFP or Ly6g-Cre-tdTomato mice during photothrombosis-induced stroke [104], and NET formation in neuroinflammation [105,106]. These imaging findings broadly correspond to recruitment/infiltration, rTEM, vascular stalling, and NET formation, thereby highlighting in vivo behavioral features that may be related to the molecularly defined neutrophil states described above. Although IVM enables the visualization of neutrophil behavior in the brain in vivo, the direct integration of transcriptomic states with these behaviors at the single-cell level remains unresolved. Thus, integrated approaches that combine IVM with transcriptomic profiling are required to clarify how distinct neutrophil states drive ischemic brain injury in vivo.

## 4. Advanced Approaches for Bridging Molecular Signatures and Cellular Behaviors

Recent developments in machine learning have provided more comprehensive views across various research fields, and these approaches have been increasingly applied in biomedical research. Integrated scRNA-seq and machine learning-based multi-omics analyses of peripheral blood from four patients with sepsis and two healthy controls identified two neutrophil clusters (clusters 1 and 2) that were markedly expanded in patients with sepsis. These clusters exhibit distinct inflammatory transcriptomic signatures, including ALPL, CD177, S100A8, S100A9, and STXBP2, suggesting a shift toward activated and pro-inflammatory neutrophil states [107]. A recent machine learning-based transcriptomic study of patients with sepsis identified S100A12 as a robust diagnostic biomarker for sepsis associated with sepsis progression and proposed it as a putative therapeutic target [108]. Consistently, in hepatocellular carcinoma, Deep scSTAR-based analysis also identified S100A12^+^ tumor-associated neutrophils that correlated with poor prognosis and immunotherapy non-response [109]. Collectively, advances in single-cell analysis combined with machine learning are expected to facilitate the efficient interpretation of large-scale multidimensional datasets and accelerate the discovery of novel therapeutic targets.

With advances in machine learning-based transcriptomic studies, recent research has enabled the high-resolution identification of disease-associated immune cell states at the molecular level. However, transcriptomic signatures cannot capture the dynamic functional states of immune cells in living tissues. In addition, cell dissociation-based scRNA-seq inevitably loses spatial and temporal context, limiting its ability to directly reflect dynamic cellular behavior in vivo [110]. To overcome these limitations, single-cell behavioral analyses obtained through intravital imaging provide a complementary framework for understanding immune cell heterogeneity in vivo. For example, individual immune cell behavioral profiling combined with computational clustering has demonstrated distinct behavioral states in inflamed tissues that are functionally associated with pathological roles in inflammatory conditions [21]. In addition, four-dimensional IVM with quantitative single-cell tracking also showed the emergence of highly migratory or adhesive αMβ2^high^ neutrophil states during ischemic stroke [22]. Nevertheless, IVM is also limited by tissue accessibility and imaging depth, as well as potential biases related to labeling strategies and imaging windows. Moreover, imaging-derived behaviors cannot be directly linked to molecular states, because these features are not measured in the same cells [111]. One complementary strategy would be to combine intravital imaging with image-guided or time- and site-matched sampling to isolate neutrophil populations enriched for tissue-damaging behaviors under inflammatory conditions, and then define their transcriptomic profiles. This strategy could help connect imaging-derived behavioral phenotypes with molecular states, thereby facilitating the identification of transcriptional features associated with pathologically relevant neutrophil populations. Collectively, the combination of machine learning-driven omics analyses with in vivo single-cell behavioral profiling offers a multidimensional approach that bridges molecular signatures and cellular behaviors, thereby enabling a better understanding of immune cell dynamics under both physiological and pathological conditions.

## 5. Concluding Remarks

Taken together, the advanced convergence of single-cell transcriptomic profiling with real-time intravital imaging provides a novel perspective on how neutrophil heterogeneity is conceptualized, from static molecular signatures to dynamic cellular behaviors embedded within tissue microenvironments. Furthermore, integrative approaches combining omics studies, high-resolution live imaging, and artificial intelligence-assisted behavioral quantification will be useful for delineating the associations between gene programs, cellular trajectories, and context-dependent immune functions. Such multidimensional frameworks are expected to not only refine mechanistic insights into neutrophil heterogeneity across physiological and pathological conditions but also enable predictive and state-specific intervention strategies. Although these approaches remain speculative, integrated molecular and behavioral analyses may help identify transcriptionally defined neutrophil states linked to pathogenic in vivo behaviors. This may support more precise immunomodulatory strategies that selectively target tissue-damaging neutrophils while preserving protective host defense functions. Ultimately, bridging molecular signatures with spatiotemporal behavior is expected to advance neutrophil research from descriptive heterogeneity to precise immunomodulation and the translational discovery of therapeutic targets under inflammatory conditions.

## Figures and Tables

**Table 1:** Surface markers and gene expression associated with neutrophil heterogeneity in cancer.

Disease	Functional Category	Surface Marker or Gene	Characteristics & Functional Outcomes	IVM-Detectable Behavior/Response	Reference
Cancer	Anti-tumor/ protective	Fpr1	associated with enhanced infiltration and cytotoxic activity of CD8^+^ and γδ T cells	Chemotaxis	[43]
		CD44^−^CXCR2^−^	reduced tumor progression and improved survival	-	[46]
		ELANE	increased CD8^+^ T cell activation	-	[47]
		HLA-DR^+^CD74^+^	exhibit an MHC class II/antigen-presenting signature and are associated with better clini-cal outcomes	-	[45]
	Immunosuppressive/tumor-promoting	Smad3	contributes to N2 polarization	-	[37]
		miR-941	promote N2-like TAN polarization and tumor progression	-	[38]
		SiglecF	N2-like features and increased ROS-related signatures	-	[41]
		CD83	the upregulation of CXCR4 and downregulation of CXCR2/SELL, consistent with a senes-cent-like phenotype and immunosuppressive signatures	-	[51]
		Nectin2	associated with CD8^+^ T-cell dysfunction/exhaustion	Cell-cell adhesion	[48]
		CXCR4^high^	characterized by ferroptotic signatures together with T-cell suppressive features	Chemotaxis	[49]
		PLAUR	reduced CD8^+^ T-cell infiltration and macrophage-driven immunosuppression	Migration & pericellular proteolysis	[50]
		platelet gene-high neutrophils	associated with degranulation, chemotaxis, and TEM	Chemotaxis and trans-endothelial migration	[52]
	Pro-inflammatory/ NET-associated	CD66b^high^CD11b^low^CD10^−^	ARG1-linked immunosuppressive/angiogenic signatures and degranulation/NETosis-like states	NETosis	[53]
		PPIF	high NET-related score, consistent with NETosis	NETosis	[54]
		CD10^+^TREM1^+^CXCR2^+^	exhibit T-cell suppressive activity and NETosis, and are associated with poor outcomes	NETosis	[55]
	Metabolic	dcTRAIL-R1	inflammation-, hypoxia-, and glycolysis-associated gene signatures	-	[56]
		MIF	associated with hypoxia- and glycolysis-related signatures	-	[57]

**Table 2:** Surface markers and gene expression associated with neutrophil heterogeneity in sepsis.

Disease	Functional Category	Surface Marker or Gene	Characteristics & Functional Outcomes	IVM-Detectable Behavior/Response	Reference
Sepsis	Anti-inflammatory/ protective	CD49c^high^ C1q^+^	associated with improved survival	Recruitment	[71]
	Immunosuppressive	IL1R2	representing an immature and immunosuppressive state linked to poor clinical outcomes	-	[76,77]
		PD-L1	suppresses T-cell activation and can additionally promote endothelial injury and pyroptosis	Endothelial injury (Vascular leakage)	[15,78,79,80,81,82]
	Pro-inflammatory/ NET-associated	OLFM4	associated with 60-day mortality	-	[72,73]
		Ly6G^+^ CD11b^high^	enhanced production of pro-inflammatory mediators and increased NET formation	Migration & NETosis	[74]
		S100A8/A9^high^	promote endothelial mitochondrial dysfunction and PANoptosis	NETosis	[75]

**Table 3:** Surface markers and gene expression associated with neutrophil heterogeneity in ischemic stroke.

Disease	Functional Category	Surface Marker or Gene	Characteristics & Functional Outcomes	IVM-Detectable Behavior/Response	Reference
Ischemic stroke	Pro-inflammatory/ tissue-damaging	mt-Nd1-4, Il1b, Aif1, and Ly86	highly activated neutrophil state infiltrating the ischemic brain	Recruitment	[97]
		CD62L^low^	enriched in oxidative stress, phagocytosis, and procoagulant features	ROS	[98]
		CXCR2^low^CD62L^low^	showed senescence, degranulation, and adhesion features linked to increased BBB permeability	Recruitment & Vascular leakage	[99]
		S100A8/A9^high^	associated with BBB disruption and increased lymphocyte recruitment	Recruitment & Vascular leakage	[101]
		CD14	accounted for approximately 80% of brain neutrophils 24 h after stroke	Recruitment	[102]

## Data Availability

No new data were created or analyzed in this study. Data sharing is not applicable to this article.

## Abbreviations

The following abbreviations are used in this manuscript:

ARG1	Arginase 1
BBB	Blood–brain barrier
CASP1	Caspase-1
CLP	Cecal ligation and puncture
CyTOF	Cytometry by time-of-flight
G-CSF	Granulocyte colony-stimulating factor
GBM	Glioblastoma
HCC	Hepatocellular carcinoma
HNSCC	Head and neck squamous cell carcinoma
IFNAR1	Interferon-α/β receptor 1
IL	Interleukin
ISG	Interferon-stimulated genes
IVM	Intravital microscopy
LAG-3	Lymphocyte activation gene 3
LPS	Lipopolysaccharide
Mac-1	Macrophage-1 antigen (integrin αMβ2; CD11b/CD18)
MHC	Major histocompatibility complex
miR	microRNA
MyD88	Myeloid differentiation primary response 88
NETs	Neutrophil extracellular traps
NPA	Neutrophil-platelet aggregates
NSCLC	Non-small cell lung cancer
OLFM4	Olfactomedin 4
PANoptosis	Inflammatory cell death pathway integrating pyroptosis/apoptosis/necroptosis
PD-1	Programmed cell death protein 1
PD-L1	Programmed death-ligand 1
PMN-MDSC	Polymorphonuclear myeloid-derived suppressor cells
rTEM	Reverse transendothelial migration
ROS	Reactive oxygen species
scRNA-seq	Single-cell RNA sequencing
TANs	Tumor-associated neutrophils
TDLN	Tumor-draining lymph nodes
TEM	Transendothelial migration
tMCAO	Transient middle cerebral artery occlusion
TLR	Toll-like receptor
UVB	Ultraviolet B
WNT	Wingless/Integrated signaling pathway
YAP/TAZ	Yes-associated protein / transcriptional coactivator with PDZ-binding motif
ZT	Zeitgeber time
